# Recessive coding and regulatory mutations in *FBLIM1* underlie the pathogenesis of chronic recurrent multifocal osteomyelitis (CRMO)

**DOI:** 10.1371/journal.pone.0169687

**Published:** 2017-03-16

**Authors:** Allison J. Cox, Benjamin W. Darbro, Ronald M. Laxer, Gabriel Velez, Xinyu Bing, Alexis L. Finer, Albert Erives, Vinit B. Mahajan, Alexander G. Bassuk, Polly J. Ferguson

**Affiliations:** 1 Department of Pediatrics, The University of Iowa, Iowa City, IA, United States of America; 2 Interdisciplinary Graduate Program in Genetics, The University of Iowa, Iowa City, IA, United States of America; 3 Division of Pediatric Rheumatology, Departments of Pediatrics and Medicine, University of Toronto and The Hospital for Sick Children, Toronto, ON, Canada; 4 Medical Scientist Training Program, University of Iowa, Iowa City, IA, United States of America; 5 Department of Ophthalmology and Visual Sciences, The University of Iowa, Iowa City, IA, United States of America; 6 Omics Laboratory, University of Iowa, Iowa City, IA, United States of America; 7 Department of Biology, The University of Iowa, Iowa City, IA, United States of America; "Centre de Recherche en Neurosciences de Lyon", FRANCE

## Abstract

Chronic recurrent multifocal osteomyelitis (CRMO) is a rare, pediatric, autoinflammatory disease characterized by bone pain due to sterile osteomyelitis, and is often accompanied by psoriasis or inflammatory bowel disease. There are two syndromic forms of CRMO, Majeed syndrome and DIRA, for which the genetic cause is known. However, for the majority of cases of CRMO, the genetic basis is unknown. Via whole-exome sequencing, we detected a homozygous mutation in the filamin-binding domain of *FBLIM1* in an affected child with consanguineous parents. Microarray analysis of bone marrow macrophages from the CRMO murine model (*cmo*) determined that the *Fblim1* ortholog is the most differentially expressed gene, downregulated over 20-fold in the *cmo* mouse. We sequenced *FBLIM1* in 96 CRMO subjects and found a second proband with a novel frameshift mutation in exon 6 and a rare regulatory variant. In SaOS2 cells, overexpressing the regulatory mutation showed the flanking region acts as an enhancer, and the mutation ablates enhancer activity. Our data implicate *FBLIM1* in the pathogenesis of sterile bone inflammation and our findings suggest CRMO is a disorder of chronic inflammation and imbalanced bone remodeling.

## Introduction

Chronic recurrent multifocal osteomyelitis (CRMO) is a rare, autoinflammatory bone disease. Its primary symptom is bone pain resulting from sterile osteomyelitis. Laboratory tests are frequently normal but can reveal mild elevation of inflammatory markers including white blood cell count, erythrocyte sedimentation rate (ESR), C-reactive protein, and tumor necrosis alpha (TNF-α) [1]. CRMO is treated with non-steroidal anti-inflammatory drugs (NSAIDs), methotrexate, sulfasalazine, bisphosphonates, TNF-α inhibitors, or occasionally by IL-1 inhibition, on an empirical basis [1].

An additional inflammatory disorder is present in approximately 25% of individuals with CRMO. Most often, the associated disease is palmoplantar pustulosis (PPP), psoriasis, or inflammatory bowel disease (IBD); within IBD, Crohn disease is the most common. These associated conditions frequently run in families affected by CRMO, as 50% of first or second degree relatives of probands have psoriasis, IBD, or another chronic immune-mediated disorder. The high prevalence of inflammatory disease in relatives suggests a strong genetic component to CRMO.

There are two rare syndromic forms of CRMO, Majeed Syndrome and Deficiency of IL-1 Receptor Antagonist (DIRA). In general, these are disorders that affect individuals at a very young age. CRMO presents before 2 years in Majeed and in infancy in DIRA [1, 2]. Majeed Syndrome is caused by homozygous mutations in *LPIN2 [3]* and DIRA is caused by recessive loss-of-function mutations in *IL1RN [4]*. Patients with both syndromes respond well to IL-1 blocking agents [5]. However, most cases of CRMO are non-syndromic and with unknown genetic cause [1, 2].

There are several mouse models of CRMO with homozygous mutations in *Pstpip2*. All models have extensive multifocal osteomyelitis and inflammation of the tissues of the ears including the skin [6–8]. PSTPIP2 is a cytoskeletally associated protein involved in membrane formation that reduces inflammation and may play a role in osteoclastogenesis [9]. Recently, it was shown that the *cmo* disease phenotype is greatly rescued by IL-1R1 deficiency, and that the NLRP3 inflammasome plays a redundant role with caspase-8 in IL-1β mediated osteomyelitis [10, 11]. The IL-1β over-secretion appears to be neutrophil driven and can be counteracted by serine protease inhibitors *in vitro [10, 12].*

Monocytes from CRMO patients have decreased IL-10 expression in response to LPS stimulation [13]. There are three SNPs in the IL-10 promoter–rs1800896, rs1800871 and rs1800872 –that compose a haplotype, and some haplotypes are significantly more common in patients compared to healthy controls [13, 14]. The haplotypes ATA and ACC are associated with relatively low and medium IL-10 expression, respectively, and the haplotype GCC is associated with high expression [14].

Here, we report on a South Asian child from a consanguineous union with CRMO. Whole exome sequencing of the child and her parents revealed a list of 23 rare, protein-changing variants in 22 genes for which the child is homozygous, having inherited one copy of the mutation from each parent. Of these 22 genes, *FBLIM1* stood out because it was the most differentially expressed gene from a microarray experiment examining macrophage expression in the *cmo* mouse. In addition, studies suggest FBLIM1 is an anti-inflammatory molecule regulated by STAT3, and one involved in bone remodeling via ERK1/2 phosphorylation and the subsequent regulation of RANKL activation. We sequenced *FBLIM1* in a larger cohort of 96 patients with CRMO and found one compound heterozygote in *FBLIM1*, with a novel frameshift insertion in exon 6 of one allele and an enhancer variant in the third intron of the other allele. The enhancer contains binding sites for STAT3 and NR4A2. NR4A2 is a transcription factor that is also involved in regulation of the inflammatory response. Enhancer activity of a 1-kb region around the mutation was validated by luciferase assays in fluoride-treated SaOS2 cells, as was the effect of the mutation on regulatory activity. Because *FBLIM1* expression is regulated by STAT3 as a function of IL-10 anti-inflammatory activity, we sequenced the SNPs in the IL-10 promoter haplotype and determined that the child with the enhancer variant is homozygous for the haplotype (ATA/ATA) that is associated with low IL-10 expression, and the child with the R38Q mutation is heterozygous (ATA/ACC). Our results implicate mutations in *FBLIM1* in CRMO and autoinflammatory disease. Mutations in *FBLIM1* likely contribute to CRMO pathogenesis epistatically with variants involved in the regulation of IL-10 expression.

## Methods

### Use of humans in research

All human research was approved by the University of Iowa Institutional Review Board (IRB). Written informed consent was obtained from all study participants and written parental or guardian consent was obtained. All participants were under the age of eighteen. Blood and saliva collected for DNA analysis were taken specifically for this study.

### Use of mice in research

Mice were used as there is no adequate *in vitro* or invertebrate animal to model autoinflammatory bone disease. All animal care, and experiments and protocols using mice were conducted in accordance with the University of Iowa Institutional Animal Care and Use Committee (IACUC). Mice are housed in cages in a Specific-Pathogen-free (SPF) barrier facility and are not housed singly. The mice breed and move about the cage without difficulty. The mice have extra bedding to make the floor of the cage soft. To minimize pain, anesthetics are used when needed, and the mice are handled gently. Each procedure was performed in a time-efficient manner and any animals that appeared to be in discomfort or ill were euthanized. Euthanasia was performed by a trained laboratory technician under guidance by a veterinarian, and mice were euthanized by 100% carbon dioxide (C0_2_) administered continuously until at least 1 minute after breathing stops followed by cervical dislocation. Mice were euthanized prior to harvesting tissue for gene expression analysis.

### Whole exome sequencing and analysis

DNA from the child and both parents was purified from saliva and prepared for whole exome sequencing. The DNA was enriched using the Agilent SureSelectXT Human All Exon V4 (Agilent Technologies) before sequencing at Otogenetics, Inc (Atlanta, GA). The fastq files were quality-checked using FastQC (http://www.bioinformatics.babraham.ac.uk/projects/fastqc/) and the raw reads were aligned to the hg19 (NCBI Build 37) reference genome using the Burrows-Wheeler Alignment (BWA) software [15] and duplicates were removed using Picard Tools. GATK software [16] was used to realign indels, for base quality recalibration and to calculate coverage. Variants were called using GATK’s UnifiedGenotyper and were recalibrated using OMNI, HapMap [17] and 1000 genomes [18] data as training sets. Variants were hard-filtered in GATK based on mapping quality (MQ>40) and depth of coverage (QD>2.0) and variants were annotated with minor allele frequencies (MAFs) from EVS (Exome Variant Server, NHLBI GO Exome Sequencing Project (ESP), Seattle, WA (URL: http://evs.gs.washington.edu/EVS/)), dbSNP [19] and ExAC [20] using GATK’s VariantAnnotator. SNPSift/SNPEff [21] was used to complete annotation from dbSNP and dbNSFP. The variants were uploaded to VarSifter [22] and non-coding variants were removed, along with those not passing hard-filtering, and variants with a MAF>2% in either the global or South Asian population. The resulting list was queried for rare homozygous variants on the autosomal chromosomes where one allele was present in each parent. Variants satisfying these criteria were validated by Sanger sequencing.

### *FBLIM1* Sanger sequencing

The coding regions of *FBLIM1* were sequenced in a cohort of 96 people with CRMO using DNA purified from either saliva or blood. The primers used to amplify each exon are listed in Table 1 below. Crude PCR products were sent to Functional Biosciences (Madison, WI) for Sanger sequencing.

**Table 1:** Primers for *FBLIM1* Sanger sequencing.

Fblim1 exon	Forward	Reverse
Exon3	ggtgctggactgagttgtt	tgtcccggaaattctgacat
Exon 4	gtgcctggcctgagattaag	cctcagagctgaaggaggtg
Exon 5	ctcggtacaatgcggctaat	tgcaggcttctcactgtgtc
Exon 6	gtgctggcattacaggtgtg	cactgggctccttctttctg
Exon 6 alternative	aagcaatggtggaggttttg	cccgagtagctgggactaca
Exon 7	tgcaggcctctcctgtactt	cactgcaaactccacctcct
Exon 8	agcccgagttctgacagcta	accatgagccaacagattcc
Exon 9	caactttccctgaaccctca	ggctcagaaggaaagtgtgc

### *IL-10* promoter Sanger sequencing

The SNPs rs1800871 and rs1800872 were sequenced via Sanger sequencing using the primers previously described: forward 5’ GACAACACTACTAAGGCTTC and reverse 5’ GCTAACTTAGGCAGTCACCT [23]. For the polymorphism rs1800896, we designed and used primers with the sequences: forward 5’ CATCAAAGGATCCCCAGAGA and reverse 3’ GGCACATGTTTCCACCTCTT.

### *IL-10* promoter haplotype assessment in 1000 genomes participants

Genotypes for the polymorphisms rs1800896, rs1800871, and rs1800872 were downloaded for all 1000 genomes [24] participants using the 1000 genomes browser. For the 489 South Asian (SAS) 1000 genomes participants, the frequencies of the ATA/ACC and ATA/ATA haplotypes were calculated.

### Gene expression microarray of *cmo* mouse bone marrow derived macrophages

Under aseptic conditions, the whole femur was collected from 4-month-old mice and stored in 1–2 ml α-MEM (serum free) ice-cold media. The whole marrow was flushed into 200μl α-MEM media in a microcentrifuge at 10000 rpm for 30s. The supernatant was removed. Marrow cell pellets were washed with 1ml α-MEM media and then centrifuged at 10000rpm for 2 minutes. The cell pellets were resuspended in 1ml media (α-MEM + 5% CMG) and cell suspensions were then transferred onto Petri dishes with a total volume of 20 ml media (α-MEM + 10% FBC + 5% CMG) for incubation at 37°C in 5% CO2 for 2 days. After a two-day incubation, the cells were harvested to undergo RNA extraction. The purification of RNA from the bone marrow macrophage cells was performed using Qiagen’s RNA purification kit (Cat#74104) according to the manufacturer’s instruction. RNA samples were sent to the University of Iowa DNA Core Facility for microarray analysis. Cells were cultured from 3 mice from each of the *cmo*, *cmo IL1R*^*+/-*^ and *cmo IL1R*^*-/-*^ strains.

RNA sample preparation and subsequent hybridization to the Illumina beadchips were performed at the University of Iowa DNA Facility using the manufacturer’s recommended protocol. Briefly, 100 nanograms total RNA was converted to amplified Biotin-aRNA using the Epicentre TargetAmp-Nano Labeling Kit for Illumina Expression BeadChip (Illumina, Inc., San Diego, CA, Cat. #TAN07924) according to the manufacturer’s recommended protocol. The amplified Biotin-aRNA product was purified through a QIAGEN RNeasy MinElute Cleanup column (QIAGEN Cat #74204) according to modifications from Epicentre. 750ng of this product were mixed with Illumina hybridization buffer, placed onto Illumina-Mouse WG-6 v2.0 BeadChips (Part No. BD-201-0202), and incubated at 58°C for 17h, with rocking, in an Illumina Hybridization Oven. Following hybridization, the arrays were washed, blocked, and then stained with streptavidin-Cy3 (Amersham/GE Healthcare, Piscataway, NJ) according to the Illumina Whole-Genome Gene Expression Direct Hybridization Assay protocol. Beadchips were scanned with the Illumina iScan System (ID #N0534) and data were collected using the GenomeStudio software v2011.1.

### Cell culture and luciferase assays

SaOS2 cells were ordered from ATCC (Manassas, VA). Cells were maintained in McCoys 5a medium + 15% FBS and 1% P/S. SaOS2 cells were stimulated to osteoblast differentiation by the addition of NaFl at a concentration of 0.2 mM to the media immediately after transfection. The *Fblim1* enhancer region was amplified by PCR and cloned into competent cells using the Gateway pENTR Directional TOPO Cloning kit from Invitrogen / Thermo Fisher Scientific (Waltham, MA). The sequences for the primers used to amplify the region are F: CTGTACCCCACTCTGTCCCT and R:GATCTGCTGAATCAGAGA. The enhancer was then recombined into the FFL Luciferase vector and amplified in Dh5α cells. The enhancer mutation rs41310367 was induced in the plasmid by site-directed mutagenesis at GenScript Inc. (Piscataway, NJ).

The Renilla and Firefly luciferase- containing plasmids were transfected into SaOS2 cells using Lipofectamine 3000 from Invitrogen / Thermo Fisher Scientific. Transfected cells were incubated for 72 hours prior to lysis. Luciferase assays were performed using the Promega (Madison, WI) Dual-Luciferase assay system. Within each experiment and treatment, there were four biological replicates. The experiment was performed three times and data from multiple experiments were combined prior to statistical analysis. Firefly to Renilla ratios were calculated for each sample and analyzed for differences between absence and presence of enhancer, and between wild-type and mutant enhancer. A two-tailed t-test was performed for each comparison.

### Multiple sequence alignment

The peptide sequence for human FBLP1 was obtained from Ensembl (www.ensembl.org), March 2016) and input into NCBI protein BLAST and queried against the non-redundant protein sequence database. Fasta sequences for *Mus musculus*, *Rattus norvegicus*, *Pan paniscus*, *Bos taurus*, *Equus caballus*, *Canis lupus familiaris* and *Felis catus* were downloaded and input into Clustal Omega [25] for multiple sequence alignment. Default settings were used to create the alignment.

### Analysis of transcription factor binding recognition sites

To predict the effect of the regulatory mutation rs41310367, we queried the sequence flanking the SNP with and without the variant for transcription factor binding site recognition motifs using the JASPAR database[26]. For the input sequences, we used the reference sequence AAGACCA**C**GTCAC, the alternative reference sequence based on conservation AAGACCA**G**GTCAC, and the variant sequence AAGACCA**T**GTCAC. Only recognition scores greater than 86 were considered for the initial analysis and then a threshold of 70 was set to determine the score after a sequence change that caused the score to be below 86.

### Structural modeling and analysis of *FBLIM1* (FBLP1)

Protein structural disorder predictions were performed using DISOPRED [27]. Secondary structure predictions were performed using PSI-PRED [28]. The tertiary structure of the FBLIM1 Filamin-1 binding domain (residues 1–70) was modeled using Phyre2 [29]. We next modeled the Filamin-1-FBLP1 complex by superimposing our Phyre2 model to NMR structure of Filamin-1 bound to a FBLIM1 peptide (PDB: 2K9U). Charges and hydrogen atoms were added to the wild-type and mutant FBLP1 models using PDB2PQR [30]. Electrostatic potentials were calculated using APBS [31]. Protein and solvent dielectric constants were set to 2.0 and 78.0, respectively. PyMOL generated all structural figures.

## Results

### Case report

**Fig 1:**
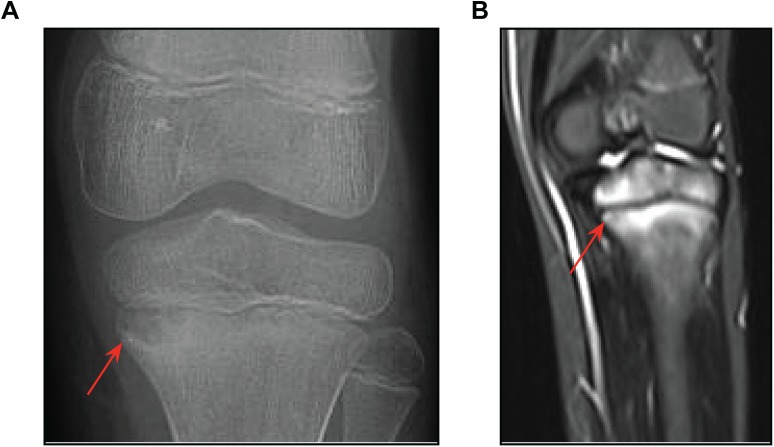
X-ray and MRI knee images from the affected proband. (A) X-ray showing osteomyelitis and a lesion characteristic of CRMO (red arrow) in the left proximal tibia. (B). MRI of the same knee showing inflammation and bone destruction (red arrow). Similar lesions were found in the clavicle, hip, femur, tibia, foot and toes (not shown). (C) MRI of a healthy knee for comparison.

An eight-year old South Asian girl was admitted to The Hospital for Sick Children (Toronto, ON) with left knee pain and an inability to bear weight. Plain x-rays were consistent with osteomyelitis of the left proximal tibia (Fig 1A). Her CBC was normal, ESR was elevated to 42 mm/hr (normal < 20), while her CRP was normal (4.2 mg/L). Cefazolin was given as therapy. An MRI of the left lower extremity showed abnormalities involving the left proximal tibial metaphysis and epiphysis and similar abnormalities in the left distal femoral metaphysis and the distal tibial metaphysis, all consistent with chronic recurrent multifocal osteomyelitis (Fig 1B). A whole body MRI showed additional lesions in the second and third right metatarsals, right cuneiform, right acetabulum, distal left humeral metaphysis and epiphysis and the left clavicle in a distribution typical of CRMO (not shown). She also had psoriasis on her right thumb and heels. Her family history was unremarkable other than that her parents were first cousins. In view of the multiple bone lesions involving metaphyses and psoriasis, a diagnosis of CRMO was made. Antibiotic therapy was discontinued. She has subsequently been treated with NSAIDs followed by two cycles of pamidronate.

### Affected child has a homozygous coding mutation in *FBLIM1*

**Fig 2:**
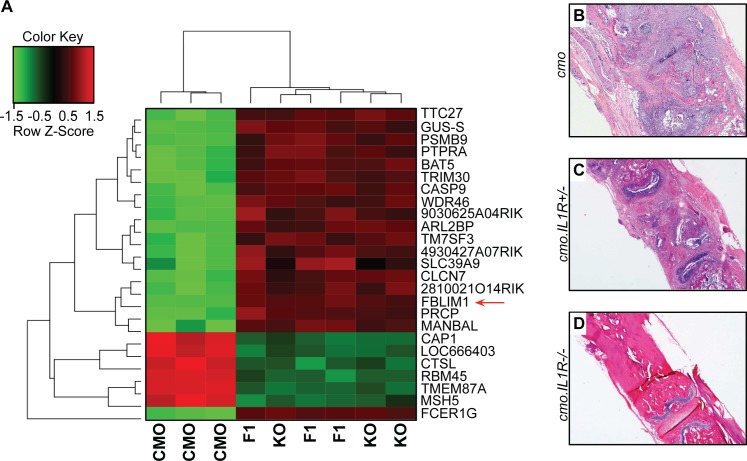
Microarray analysis of bone marrow macrophages from the *cmo* mouse model. (A) Gene expression heatmap from microarray analysis of RNA from bone marrow-derived macrophages of *cmo*, IL-1R^-/-^ and IL-1R^+/-^ mice (n = 3 for each strain). Normalized expression values were analyzed for differential expression using Partek Genomics Suite software (version 6.12, Partek Inc., St. Louis, MO, USA.). The top 25 differentially expressed genes are shown. *Fblim1* (red arrow) was the most differentially expressed (downregulated) gene in the *cmo* mouse. (B, C and D) Representative fixed decalcified tail bone sectioned and stained with H&E from *cmo*, IL-1R^-/-^ and IL-1R^+/-^ mice, respectively. The cmo mouse has extensive mixed inflammatory infiltrate with destruction of the vertebral body, the cmo.IL-1R^+/-^ has similar but less severe inflammation and bone destruction, whereas, the cmo.IL-1R^-/-^ mouse has normal bone with no inflammation. Representative tail kinks and foot deformities in these mice were depicted and described previously [10].

**Fig 3:**
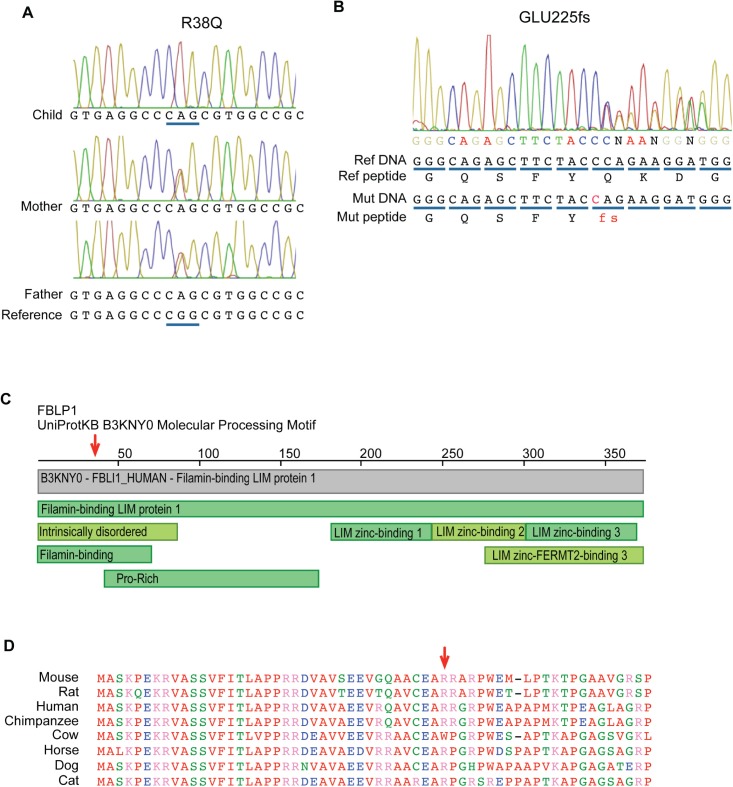
Coding mutations found in individuals with CRMO. (A) Chromatogram showing the homozygous mutation in exon 3 of FBLIM1 in a child from a consanguineous union. The mutation is rs146575757, a G to A nucleotide change, causing an Arg38Gln change in the protein. (B) Chromatogram showing a novel 1-bp frameshift insertion in exon 6 of FBLIM1 in a second proband with CRMO. The mutation is one allele of a compound heterozygote and the frameshift occurs at Glu255. (C) Image of conserved domains in FBLP1 from the Protein Data Bank (PDB) (www.rcsb.org) [32]. The red arrow points to the location of the Arg38Gln position, in the filamin-binding domain. (D) Protein alignment of FBLP1 and its orthologues in other mammals. The red arrow points to the amino acid disrupted by rs146575757.

Whole exome sequencing followed by Sanger sequencing validation revealed that the child harbored homozygous coding variants with a minor allele frequency in global or South Asian populations of less than 2% in 22 genes. The Agilent v4 platform was covered by an average number of 24.9 reads. The affected genes are listed in Table 2. Of these 22 genes, *FBLIM1* was the most promising candidate based on its known function and the results of the mouse microarray experiment (see Fig 2). Her *FBLIM1* variant consists of a G to A mutation that induces an arginine to glutamine amino acid change at position 38 (R38Q) in all known transcripts. The mutation is located in the filamin-binding domain and the affected residue is largely conserved in mammals (Fig 3). According to ExAC [20] (which does contain individuals with inflammatory diseases), this variant has a global MAF of 0.004193 and in the South Asian population, an MAF of 0.01417. Fig 3 shows the protein location of the *FBLIM1* mutation as well as a multiple sequence alignment for the surrounding region.

**Table 2:** Rare homozygous coding variants in the affected child.

Chr	bp (hg19)	Gene name	dbID	REF^a^	VAR^b^	Amino Acid Change^c^	ExAC^d^ Global	ExAC SAsian	1KG ^e^ Global	1KG SAS	Child	Father	Mother
1	16069525	TMEM182	rs41268336	C	T	Arg58Trp	0.00963	0.01592	0.006	0.008	TT	CT	CT
1	16091591	FBLIM1	rs146575757	G	A	Arg38Gln	0.00419	0.01417	0.004	0.009	AA	AG	AG
1	26524551	CATSPER4	rs560352853	A	G	Ile221Val	0.00202	0.01429	0.003	0.016	GG	AG	AG
1	230824171	COG2	rs201551743	C	G	Leu553Val	9.06E-05	0.00061	0	0.001	GG	CG	CG
3	196742243	MFI2	rs45625439	C	T	Arg409Gln	0.02026	0.00867	0.005	0.007	TT	CT	CT
6	118953690	CEP85L	rs560509236	T	C	His56Arg	0.00115	0.00806	0.002	0.008	CC	CT	CT
7	48317939	ABCA13	rs200243325	T	G	Ile2383Arg	0.00197	0.00997	0.001	0.005	GG	GT	GT
8	10469719	RP1L1	-	G	A	Thr630Ile	-	-	-	-	AA	AG	AG
8	10480645	RP1L1	rs188979626	G	A	Arg23Cys	1.79E-05	0	0	-	AA	AG	AG
12	12966323	DDX47	rs577622688	G	T	Asp8Tyr	0.00158	0.01089	0.002	0.009	TT	GT	GT
12	54118405	CALCOCO1	rs373349679	C	A	G95*|	0.00322	0.01715	0.002	0.008	AA	AC	AC
14	69259681	ZFP36L1	rs139683444	C	T	Arg61His	0.00018	0.00097	0	-	TT	CT	CT
15	90610517	ZNF710	rs144139243	G	C	Glu50Gln	0.00239	0.01305	0.001	0.005	CC	CG	CG
16	72822645	ZFHX3	rs149133285	G	A	Ser3177Leu	0.00699	0.01757	0.005	0.016	AA	AG	AG
19	13941205	ZSWIM4	rs372292857	G	A	Ala771Thr	0.00033	0.00237	0	0.002	AA	AG	AG
19	17638144	FAM129C	rs200304763	A	C	Ser30Arg	0.00532	0.01283	0.001	0.006	CC	AC	AC
19	19648290	YJEFN3	rs547825267	G	A	Arg286His	0.00105	0.00565	0.001	0.003	AA	AG	AG
19	23927685	ZNF681	-	C	G	Glu223Gln	-	-	-	-	GG	CG	CG
20	57599401	TUBB1	rs62639974	C	T	Arg307Cys	0.00443	0.00515	0.011	0.002	TT	CT	CT
20	55027781	CASS4	-	C	T	Arg517Trp	-	-	-	-	TT	CT	CT
20	61288068	SLCO4A1	-	T	C	Trp88Arg	-	-	-	-	CC	CT	CT
22	43213187	ARFGAP3	rs144427016	T	C	Asn342Ser	0.00121	0.00798	0.001	0.006	CC	CT	CT
22	44515711	PARVB	rs742550	G	A	Arg140His	0.01606	0.01331	0.008	0.007	AA	AG	AG

^a^REF = reference allele

^b^VAR = variant allele

^c^Amino acid position refers to canonical transcript, Minor allele Frequencies

^d^ExAC = Exome Aggregation Consortium[20]

^e^1KG = 1000 genomes[18].

### *FBLIM1* is the most differentially expressed gene in BMM from *cmo* mice

We examined *FBLIM1* expression in the *Pstpip2-*null *cmo* mouse model. These mice are protected from disease on an *IL-1R*-deficient genetic background, and disease is delayed in a heterozygous background (appearing at 21 weeks vs. 8 weeks in the *cmo* mouse) [10]. To assay gene expression in these mice, bone marrow derived macrophages from *cmo*, *cmo IL-1R*^*+/-*^ and *cmo IL-1R*^*-/-*^ four-month olds were harvested and cultured for microarray analysis. Notably, *Fblim1* was the most differentially expressed gene in the *cmo* mouse, and was downregulated 26.3-fold (p = 7.74 x 10^−9^) compared to *cmo IL-1R*^*+/-*^, and downregulated 21.4-fold (p = 1.14x10^-8^) compared to *cmo IL-1R*^*-/-*^ (Fig 2A). The top 25 differentially expressed genes are listed in Table 3.

**Table 3:** Top 25 differentially expressed genes in the mouse *cmo* microarray experiment.

	CMO vs. IL1R^+/-^			CMO vs. IL1R^-/-^		
Gene	p-value	Fold-Change	Effect in CMO	p-value	Fold-Change	Effect in CMO
*FBLIM1*	7.74E-09	-26.3414	down	1.14E-08	-21.4121	down
*FCER1G*	3.76E-08	-768.539	down	3.47E-08	-840.44	down
*TMEM87A*	1.25E-07	33.9359	up	1.08E-07	37.0694	up
*CASP9*	5.06E-07	-10.0818	down	5.33E-07	-9.88089	down
*ARL2BP*	5.93E-07	-6.58926	down	6.11E-07	-6.52675	down
*GUS-S*	7.42E-07	-14.3302	down	1.06E-06	-12.2753	down
*LOC666403*	8.88E-07	40.3891	up	1.31E-06	31.8832	up
*TTC27*	1.06E-06	-15.8034	down	9.23E-07	-16.8946	down
*BAT5*	1.32E-06	-21.3405	down	1.04E-06	-24.2149	down
*MSH5*	1.24E-06	4.01022	up	2.74E-06	3.36972	up
*4930427A07RIK*	1.68E-06	-3.11473	down	4.04E-06	-2.6625	down
*WDR46*	2.66E-06	-11.3828	down	2.52E-06	-11.6345	down
*RBM45*	2.52E-06	30.9757	up	2.86E-06	28.8087	up
*CAP1*	2.78E-06	19.3367	up	3.35E-06	17.6384	up
*PRCP*	3.23E-06	-10.3049	down	5.24E-06	-8.56969	down
*9030625A04RIK*	3.65E-06	-11.273	down	1.08E-05	-7.49047	down
*TRIM30*	4.63E-06	-22.0663	down	5.75E-06	-19.7381	down
*2810021O14RIK*	4.91E-06	-5.91873	down	6.63E-06	-5.41564	down
*PSMB9*	5.80E-06	-10.1156	down	6.32E-06	-9.78298	down
*CLCN7*	5.90E-06	-4.40784	down	7.57E-06	-4.14429	down
*TM7SF3*	7.55E-06	-8.13616	down	4.36E-06	-9.99593	down
*CTSL*	7.24E-06	5.25264	up	1.05E-05	4.74202	up
*MANBAL*	9.29E-06	-4.12521	down	1.69E-05	-3.59344	down
*SLC39A9*	9.30E-06	-2.46427	down	0.000132813	-1.7638	down

### FBLP1 structural modeling

**Fig 4:**
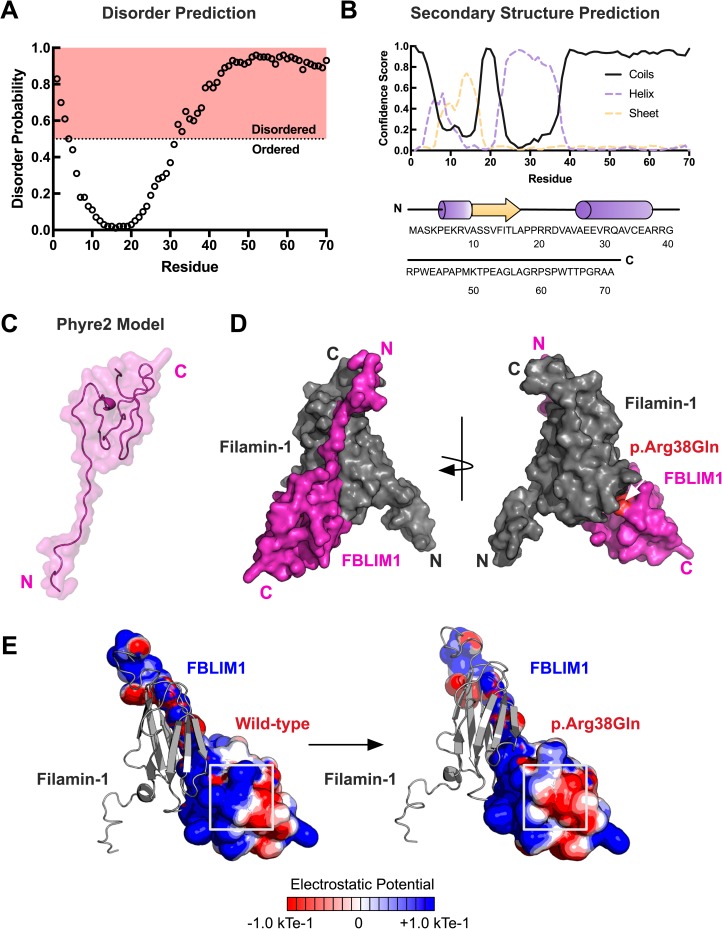
Structural modeling of patient mutation in *FBLIM1*. **(**A) Disorder prediction of the Filamin-1 binding region of FBLP1 (residues 1–70) was performed with DISOPRED version 3. Disorder probability scores greater than 0.5 are considered to be disordered. The R38Q mutation falls in a predicted disordered region. (B) Secondary structure prediction was performed using PSI-PRED. The confidence scores for coils, helices, and strands are shown. (C) The output model of the Filamin-1 binding region of FBLIM1 generated in Phyre2. A total of 86% of the residues are predicted to be disordered, resulting in 76% of the residues having a low confidence score (<90%). Residues 1–24 are predicted to be ordered. (D) Superimposition of the Phyre2 model onto the NMR structure of the Filamin-1-FBLIM1 complex. Modeling of the complex places the R38Q mutation downstream of the interaction site. (E) Electrostatic potential calculations in APBS reveal a loss of positive charge in FBLP1 as a result of the CRMO mutation.

Previous structural studies on FBLP1 using nuclear magnetic resonance (NMR) spectroscopy have determined its structure in complex with Filamin-1 [33]. However, these structures do not cover the region that is mutated in our patient. Therefore, we attempted to model the full Filamin-1 binding region of FBLIM1 using an *ab initio* approach in the Phyre2 program [29]. First, we submitted the primary structure to the DISOPRED and PSI-PRED servers [27, 28]. These programs predicted the Filamin-1 binding region to be highly disordered and mostly coiled (Fig 4A and 4B). Similarly, the resulting model from Phyre2 was predicted to be 86% disordered (Fig 4C). The ordered residues (positions 1–24) corresponded directly with the previously-determined NMR structure of FBLP1 (PDB: 2K9U). The model was in agreement with our primary structure analysis that predicted the Filamin-1 binding domain to be an intrinsically-disordered region. Superimposition of our model onto the previously-determined Filamin-1 complex revealed the R38Q mutation to be downstream of the Filamin-1 interaction site (Fig 4D).

Intrinsically-disordered regions (IDRs) in proteins are known to be important for interactions with other proteins and binding partners [34]. Since the Filamin-1-binding domain was predicted to be intrinsically-disordered, we sought to determine other potential deleterious effects of the R38Q mutation on FBLP1 function other than conformational changes. The substitution from an arginine to a glutamine removes a positive charge downstream of the Filamin-1 interaction site. This resulting change in electrostatic potential was modeled using APBS (Fig 4E) [31]. Taken together, our structural modeling results place the patient mutation downstream of the Filamin-1 interaction site on an intrinsically-disordered region and predict a loss of positive charge, which may alter FBLP1’s interaction with Filamin-1. Since the Filamin-1-binding region of FBLP1 is predicted to be an IDR, the results of our structural modeling are limited and require further biophysical studies to determine the effects of CRMO mutations on FBLP1 structure and function.

### *FBLIM1* sequencing in our CRMO cohort

**Fig 5:**
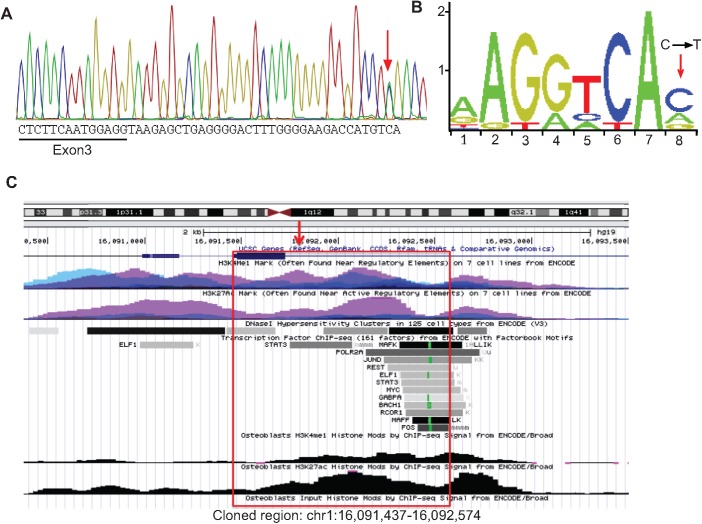
*FBLIM1* enhancer variant in 2^nd^ proband. (A) Chromatogram from the second proband. Red arrow indicates rs41310367 (C→T) in the third intron of *FBLIM1*. (B) From JASPAR [26], the human NR4A2 consensus transcription factor binding site (TFBS) motif. The red arrow shows where rs41310367 is located within the binding site and that the TFBS is disrupted. (C) Screen capture from UCSC genome browser (hg19) showing the regulatory region that includes rs41310367 (red arrow) and a STAT3 binding region. H3K27ac and H3K4me1 peaks from ENCODE are shown, including in osteoblasts. The red box indicates the region that was amplified and cloned into the Firefly reporter vector.

*FBLIM1* was sequenced in a cohort of 96 unrelated individuals with CRMO. All rare (global MAF < 2%), coding variants detected are summarized in Table 4. Among this group was a second child, also of South Asian ancestry, who harbored two FBLIM1 mutations: a novel 1-bp frameshift insertion in exon 6, (the fourth coding exon, Fig 2B) and a variant (rs41310367) in an intron, 32 nucleotides 3’ of the end of the first coding exon (Fig 5A). Sequencing the parents verified that the child inherited one mutation from each parent. According to data from the ENCODE project, the intronic variant (rs41310367) is centrally located in the middle of an enhancer, in a STAT3 binding region and in an NR4A2 recognition site (Fig 5B and 5C). The enhancer was reported to be active in several cell lines, including osteoblasts, since it was covered by H3K27ac and H3K4me1 peaks, the two marks of an active enhancer [35] (Fig 5C). Chromatograms for the *FBLIM1* mutations found in the family with the R38Q mutations and the family with the frameshift and rs41310367 mutations are provided in the S1 Chromatograms.

**Table 4:** Rare (<2% GMAF^a^) mutations^b^ found in *FBLIM1* in a cohort of 96 subjects with CRMO.

Subject (total number)	exon3^c^	rs41310367	exon4	exon5	exon6	exon7	exon8	Reported Ancestry
1–72^d^ (72)	x^e^	X	x	x	x	x	x	European
73–76 (4)	X	yes^f^	x	x	x	x	x	European
77 (1)	X	X	x	x	x	n/a ^g^	x	European
78 (1)	X	X	x	n/a	x	x	x	European
79–88 (10)	X	X	x	x	x	rs114077715	x	European
89 (1)	X	X	x	x	x	x	x	Latin American
90 (1)	X	Yes	x	x	Glu225fs	x	x	South Asian
91–94 (4)	X	X	x	x	x	x	x	East Asian
95–96 (2)	X	X	x	x	x	x	x	Ashkenazi Jewish

^a^GMAF = global minor allele frequency.

^b^All mutations in the table listed are non-synonymous except for rs41310367.

^c^Exons 1 and 3 are 5’UTR, so they were not sequenced.

^d^Subjects pooled together are identical in *FBLIM1* genotype and reported ancestry.

^e^x = successful Sanger sequencing, no rare coding variants.

^f^yes = presence of mutation.

^g^n/a = Sanger sequencing for the exon in that individual failed.

While we did not find more individuals with homozygous or compound heterozygous mutations in *FBLIM1* in the cohort of 96 patients, there was significant enrichment for rs114077715, a non-synonymous SNP in exon 7 (Table 4). The Gly311Arg polymorphism has a global and European MAF of 0.01977 and 0.02638, respectively (ExAC). Ten of the 88 European-American individuals with CRMO carried the minor allele (MAF = 0.057), indicating two-fold enrichment compared to the European MAF (p = 0.0120). rs114077715 was not detected as part of a compound heterozygote–however, its enrichment suggests that there could be other non-coding regulatory variants like rs41310367 in *FBLIM1* contributing to the pathogenesis of CRMO.

### rs41310367 disrupts an NR4A2 recognition site

The sequence surrounding variant rs#41310367, located in the third intron of *FBLIM1* was analyzed using the JASPAR transcription factor (TF) database (JASPAR CORE Vertebrate data set) [26]. Only four candidate TFs scored higher than the 86% score threshold, including NR4A2, a C4 zinc finger class nuclear receptor. Analysis of this sequence window featuring the rs#41310367 (T) variant resulted in reduced scores for three of these TFs except for MEIS1, which was substantially increased. This same sequence window features a G nucleotide in many other species based on conservation of the regulatory region surrounding and including the base impacted by rs#41310367, as viewed in UCSC Genome Browser’s Vertebrate Multiz Alignment and Conservation track [36]. Analysis of this sequence showed that only NR4A2 was predicted to retain a high quality binding site. The scores for each of the five TFs are listed in Table 5.

**Table 5:** NR4A2 is a candidate transcription factor affected by the variant rs#.

Factor	AAGACCACGTCAC	AAGACCATGTCAC	AAGACCAGGTCAC
NR4A2	0.876	0.762	0.820 (0.866)
ATF1	0.910	0.779	0.759
ZNF354C	0.887	0.712	0.755
MEIS1	0.906	0.970	0.757 (0.702)
RORA	0.743	0.743	0.865

The five transcription factors (TFs) that recognize the region surrounding rs#41310367 are in the first column. The second column lists the JASPAR [26] scores for the reference sequence in humans. The third column lists the scores for the sequence with the minor T allele, and the last column lists the scores with the G variant at the same position, which is the consensus sequence in many vertebrates.

### *IL-10* promoter haplotype investigation

The *IL-10* promoter haplotype associated with differential *IL-10* expression contains the polymorphisms rs1800896 (G/A), rs1800871 (C/T) and rs1800872 (C/A). We sequenced the SNPs in the two families with the *FBLIM1* mutations. Phasing of each variant was validated by sequencing the SNPs in the parents. The child with the R38Q mutation is heterozygous for the low and medium expressing haplotypes ATA/ACC and the child with rs114077715 is homozygous for the lowest-expressing ATA haplotype. We investigated the frequency of the ATA/ACC and ATA/ATA haplotypes in the South Asian 1000 genomes participants and determined that the two genotypes occur at a frequency of 24.5% and 22.1% respectively. The genotypes in both children are supportive of additive effects of the *IL-10* regulating promoter haplotype and the *FBLIM1* mutations. The chromatograms for the *IL-10* promoter haplotype sequencing are provided in the S2 Chromatograms.

### Functional validation of rs41301367

**Fig 6:**
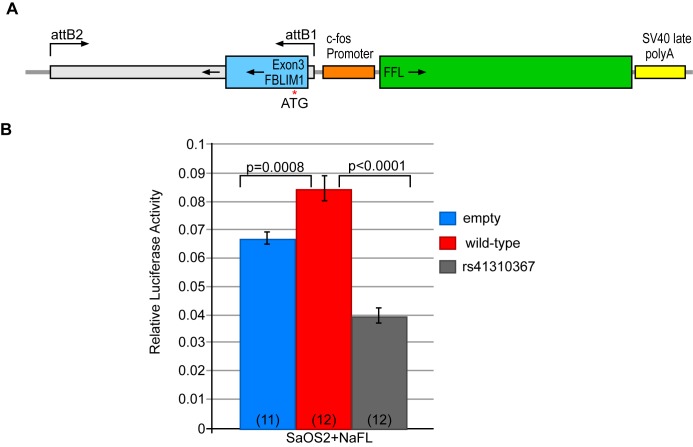
Functional validation of rs41310367 by luciferase assay. (A) Diagram of the firefly luciferase reporter driven by the *cfos* promoter plus the putative *FBLIM1* enhancer region (hg19:chr1:16,091,437–16,092,574). attB1 and attB2 are the recombination sites for the Gateway recombination system. The diagram shows recombination inserted the regulatory region into the construct backwards. The red arrow shows the position of rs41310367. (B) Firefly to Renilla luciferase ratios in transfected SaOS2 cells (72-hours after transfection). The number of replicates for each experiment is indicated in parentheses. The presence of the enhancer (red) increased luciferase activity compared to the empty vector (blue), and the mutation (gray) significantly reduced enhancer activity, both with and without fluoride treatment; however, the effects were greater and more significant in NaFl-treated SaOS2 cells. In all experiments, luciferase activity was normalized to the co-transfected Renilla luciferase. Data from individual experiments were combined prior to statistical analysis and differences in relative luciferase activity between experimental scenarios were determined using a two-tailed t-test.

The 1000 genomes global and South Asian minor allele frequencies for rs41310367 are 0.014 and 0.040, respectively, so the variant is relatively common. However, since the second proband also harbored a novel frameshift on the other *FBLIM1* allele, we functionally characterized the intronic variant in a firefly luciferase reporter construct. The 1138 base pairs flanking the SNP was cloned upstream of the *cfos* promoter, in an enhancer position (Fig 6A). Transfected cells were assayed in the presence of added fluoride, since supplemental fluoride can differentiate SaOS2 cells into osteoblasts and increase expression of osteoblast marker genes [37]. In its normal form, the 1138 bp sequence showed robust enhancer activity in the NaFl-treated SaOS2 cells, and introducing the mutation ablated this activity (Fig 6B). The raw luciferase readings are provided in S1 Table.

## Discussion

CRMO is a rare pediatric autoinflammatory bone disease and family pedigrees suggest a strong genetic component. Via whole-exome sequencing, a child from a consanguineous union affected by CRMO was found to harbor a rare, homozygous coding mutation in *FBLIM1*, and another child with CRMO was found to be a compound heterozygote for mutations in *FBLIM1*, with one frameshift mutation and one expression-altering enhancer mutation.

*FBLIM1* codes for Filamin-binding LIM protein 1 (FBLP1) or migfilin, a filamin-binding protein involved in the regulation of bone remodeling [38]. The protein has an N-terminal filamin-binding domain that binds to Filamin A/B/C, a central proline-rich domain that interacts with vasodilator-stimulated phosphoprotein (VASP), and a C-terminal LIM domain that interacts with the transcription factor CSX/NKX2-5 or mitogen-inducible gene 2 (MIG2), depending on the splice variant [39].

FBLP1 is a key regulator of the cytoskeleton, where it anchors cell-extracellular matrix adhesion proteins and filamin-containing actin filaments [39, 40]. FBLP1 binds filamin at cell-cell and cell-ECM contacts [41]. There, it competes with integrin β for filamin binding to promote integrin activation [33, 42]. This mechanism was recently demonstrated in neutrophils and vascular and endothelial cells [42]. In relation to the development of CRMO, this is very interesting in light of the recent work demonstrating macrophage-1 (mac-1) activation in neutrophils during sterile inflammation [43]. Mac-1 is composed of both integrin α and β, the latter of which interacts directly with filamin [44]. Notably, the mutation found in the child from the consanguineous family is in the filamin-binding domain of FBLP1 and so may disrupt FBLP1-FLN binding resulting in aberrant integrin activation in neutrophils. In the *cmo* mouse, the phenotype is driven by neutrophils that hyperexcrete IL-1β [10, 11].

There is a structural model of the FBLP1-Filamin1 complex based on NMR spectroscopy [33]. Unfortunately, because the R38Q mutation is outside the region included in the model, our *in silico* predictive capacity of the mutation’s effect on Filamin binding is limited. However, the R38Q mutation induces a loss of positive charge in an intrinsically disordered region; such regions are known to be involved in interacting with and/or binding to other proteins [34]. Future experiments include those that test the hypothesis that the R38Q disrupts filamin binding and subsequent integrin β activation, thereby leading to sterile inflammation.

Mice null for *FBLIM1* have severe osteopenia and increased osteoclast differentiation marked by increased RANKL expression in bone marrow stromal cells [38]. RANK/RANKL/OPG signaling was identified in the late 1990s, and in the last fifteen years, much has been discovered regarding its involvement in bone remodeling and its dysregulation in immune-mediated diseases [45–47]. RANKL/RANK binding triggers osteoclast differentiation and activation and too much of either results in excess bone resorption leading to osteoporosis and other bone disorders [46, 47]. In rheumatoid arthritis, *RANKL* overexpression in the synovial fluid is induced by Th17 signaling [48]. In *Fblim1*-null mice, increased RANKL is mediated by ERK1/2 phosphorylation and blocking ERK1/2 phosphorylation in the mice restored lower RANKL levels [38]. ERK phosphorylation is crucial for priming of the inflammasome by lipopolysaccharide (LPS) [49], suggesting that FBLP1 may act as an inhibitor of inflammasome activation.

As part of the anti-inflammatory response, *FBLIM1* is regulated by STAT3 upon macrophage stimulation by IL-10 [50]. A 2012 study identified the genomic regions bound by STAT3 and the nearby (within 20 kb) genes up- or down-regulated in macrophages treated with IL-10, an anti-inflammatory cytokine [50]. *FBLIM1* was one of the most significantly upregulated genes providing additional evidence that FBLP1 is a molecule with anti-inflammatory activity.

Given its documented role as an anti-inflammatory molecule involved in bone remodeling, *FBLIM1* was the most robust candidate gene found in the consanguineous family. Further support for *FBLIM1* came from a microarray experiment with the *cmo* mouse model of CRMO. Differential expression analysis among bone-marrow derived macrophages from the *cmo* mouse, a *cmo IL-1R*^*-/-*^ knockout and a *cmo IL-1R* ^*-/+*^ mouse found *Fblim1* to be the most significantly differentially expressed gene, downregulated over 20-fold. Sequencing the coding portion of *FBLIM1* in a larger cohort of CRMO subjects found a second proband with a novel frameshift (and likely loss-of-function) insertion in the sixth exon of *FBLIM1*, along with a variant in a putative enhancer. While we did not find more than two individuals harboring homozygous or compound heterozygous *FBLIM1* mutations, we did find two-fold enrichment for rs114077715, a coding mutation in the 7^th^ exon. This significant association coupled with our functional validation of the regulatory mutation rs41310367 suggests that there may be other non-coding mutations in linkage disequilibrium with rs114077715 contributing to CRMO disease.

*FBLIM1* is crucial for bone remodeling, which requires a balance between osteoclast and osteoblast activity. Our experimental results suggest rs41310367 disrupts this balance. More specifically, the region flanking the mutation acts as an enhancer in osteoblasts and our experiment shows that its regulatory activity is markedly ablated when the mutation is present. This implies that the mutation would enhance osteoclastogenesis and diminish osteoblastogenesis, resulting in excessive bone resorption. Our interpretation is supported by findings in the *Fblim1*-null mouse, which has decreased osteoblast and increased osteoclast differentiation [38].

The STAT3 transcription factor participates in the anti-inflammatory response mediated by IL-10 in macrophages, and in MCF10A cells, ENCODE ChIP-Seq experiments identified a STAT3 binding region at rs41310367 [51–53]. Such binding would likely enhance *FBLIM1* expression in osteoblasts. Although the transcription factor binding profile database JASPAR [26] indicates the mutation is not in the sequence recognized by STAT3, it could nevertheless disrupt binding and regulation by other transcription factors that cooperate with STAT3. That other factor may be NR4A2, since the mutation rs41310367 disrupts an NR4A2 binding site.

We tested *in silico* the effect of the regulatory variant on transcription factor (TF) binding recognition as predicted using JASPAR [26]. Recognition scores were determined using the two reference alleles for rs41310367 (C or G) among vertebrates as well as the variant allele (T). Only NR4A2 recognized the sequences featuring both the C or G nucleotides, suggesting that in the most conserved model, NR4A2 is the TF that is most likely impacted by the variant. Notably, the MEIS1 recognition score increased with the T allele. A second non-exclusive interpretation is that the variant nucleotide creates a MEIS1 binding site that competes with NR4A2 binding. However, given that NR4A2 is directly involved in the differentiation of osteoblasts [54, 55], we believe that it is a more likely candidate for involvement of the regulation of FBLIM1 expression in the fluoride-treated SaOS2 cells.

NR4A2 is an orphan nuclear receptor and transcription factor well characterized in its involvement in dopaminergic neuron signaling [56]. NR4A2 is also implicated in the inflammatory response in relation to the development of arthritis, atherosclerosis and psoriasis [56]. Its expression is significantly higher in the synovial fluid of patients with rheumatoid arthritis and in psoriatic skin [56, 57] and this overexpression is abrogated by treatment with dexamethasone, methotrexate, or by TNF-α inhibition [56, 57]. In synovial tissue, NR4A2 activates the expression of the inflammatory chemokine IL-8, [57] and NR4A2 expression and correlated TNF-α levels are modulated by thrombospondin-1 (TSP-1), [58] an ECM glycoprotein that binds to integrins and integrin-associated proteins in its role as a regulator of cell-cell and cell-ECM interaction [59]. In human chondrocytes, histamine (which induces cartilage destruction) increases RANKL activity via regulation by NR4A2 [60]. Recently, NR4A2 was identified as an NLRP3 inflammasome activation-responsive “hub” gene in human monocytes, as a transcription factor that activates the expression of anti-inflammatory genes [61]. Based on NR4A2’s documented involvement in the inflammatory response in the skin, joints, and cartilage, rs41310367 may disrupt *FBLIM1*’s regulation by NR4A2 as part of the inflammatory or anti-inflammatory response, depending on the cell type.

It is notable that aside from the novel frameshift mutation detected in the second family, the other mutations described in this report are not extremely rare. The homozygous mutation rs146575757 found in the child with consanguineous parents has a South Asian minor allele frequency of 0.01417 (ExAC) and there are two homozygotes in the ExAC database. The regulatory variant rs41310367 is even more common, with a minor allele frequency of 0.04 in the South Asian individuals in 1000 genomes, and there is one homozygote for this variant in the database. Of the 97 CRMO subjects for which *FBLIM1* was sequenced (including the child from the first family), only two harbored rare, recessive mutations, and they were the only two individuals with South Asian ancestry. It is possible that there are additional variants contributing to disease pathogenesis; in the two South Asian individuals, those variants could be common, and possibly ancestry-specific.

Given that *FBLIM1* expression is regulated by STAT3 in the context of IL-10 signaling and the IL-10 promoter haplotypes are associated with variable IL-10 expression [13, 14], we sequenced the haplotypes in the two families described in this report. Both children have haplotypes associated with relatively low *IL-10* expression; the child with the R38Q mutation is heterozygous for the ATA and ACC haplotypes and the child with the novel frameshift mutation *in trans* with the regulatory variant rs41310367 is homozygous for the lowest IL-10 expressing ATA haplotype. The ATA/ACC and ATA/ATA genotypes occur at a frequency of 24.5% and 22.1% respectively in the 1000 genomes South Asian (SAS) participants, so these probabilities multiplied by the probabilities of the *FBLIM1* mutations found in the two families result in combined genotype frequencies that are more consistent with the incidence of CRMO. The low *IL-10* expression likely contributes to loss of *FBLIM1* function in an additive manner, especially in the child with the rs41310367 variant, as low *IL-10* expression likely induces low *FBLIM1* expression synergistically with the reduced enhancer activity caused by the regulatory variant. *In trans* with the frameshift mutation, this would result in a complete loss of function in *FBLIM1*.

**Fig 7:**
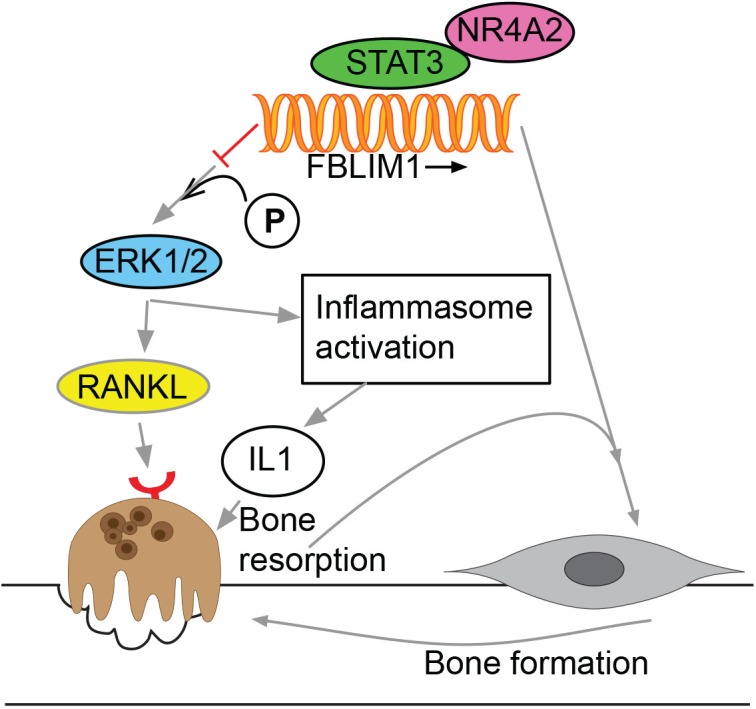
Proposed model for FBLIM1’s involvement in CRMO. *FBLIM1* expression is regulated by STAT3 and NR4A2 binding. FBLP1 blocks the phosphorylation of ERK1/2; in its absence or when dysfunctional, the increased phosphorylation of ERK1/2 leads to increased RANKL production, subsequent osteoclast activation, and bone resorption, as well as inflammasome activation. Mutations like rs41310367 disrupt regulation and expression of *FBLIM1*, resulting in decreased osteoblast activity and subsequent bone loss.

We report here that recessive mutations in *FBLIM1* contribute to the pathogenesis of chronic recurrent multifocal osteomyelitis. Previous experiments support FBLP1’s function as an anti-inflammatory molecule, and one involved in the regulation of integrin activation in neutrophils as well as the maintenance of bone homeostasis. In this study, we identified two families affected by CRMO with likely pathogenic mutations in *FBLIM1*. The results of the *cmo* mouse microarray experiment strongly support *FBLIM1* as the most likely candidate gene underlying CRMO in these two families. This is the first report of *FBLIM1* as a gene mutated in human disease. Although further experimentation is necessary to determine the exact mechanism by which aberrant *FBLIM1* function leads to the CRMO phenotype, we hypothesize that *FBLIM1* expression is regulated by STAT3 and NR4A2 in immune and bone cells and that it maintains the balance between osteoclast bone resorption and osteoblast bone formation in healthy individuals via regulation of ERK1/2 phosphorylation and subsequent RANKL expression (Fig 7). FBLP1 activity is part of the anti-inflammatory response and mutations in *FBLIM1* lead to the chronic inflammation and bone lesions characteristic of CRMO.

## Supporting information

S1 Chromatograms *FBLIM1* Sanger sequencing chromatograms. Sanger sequencing chromatograms showing that in the family (FamA) with rs146575757, the affected child is homozygous and both parents are heterozygous. For the second family (FamB), the chromatograms show the novel frameshift mutation and rs41310367, and that the mutations are *in trans*. C, D, M = child, dad, mom
(ZIP)

S2 Chromatograms *IL10A* promoter haplotype Sanger sequencing chromatograms. Sanger sequencing chromatograms showing values for rs1800896 (G/A), rs1800871 (C/T) and rs1800872 (C/A) in families A and B. C, D, M = child, dad, mom.
(ZIP)

S1 Table Raw luciferase readings. Luciferase readings from the experiments functionally validating the regulatory effect of rs41310367. The readings are shown as three separate experiments, and combined for statistical analysis.
(XLSX)
