# Correction: Recessive coding and regulatory mutations in *FBLIM1* underlie the pathogenesis of chronic recurrent multifocal osteomyelitis (CRMO)

**DOI:** 10.1371/journal.pone.0181222

**Published:** 2017-07-07

**Authors:** Allison J. Cox, Benjamin W. Darbro, Ronald M. Laxer, Gabriel Velez, Xinyu Bing, Alexis L. Finer, Albert Erives, Vinit B. Mahajan, Alexander G. Bassuk, Polly J. Ferguson

Panel C is missing from Fig 1. Please see the correct Fig 1 here.

**Fig 1:**
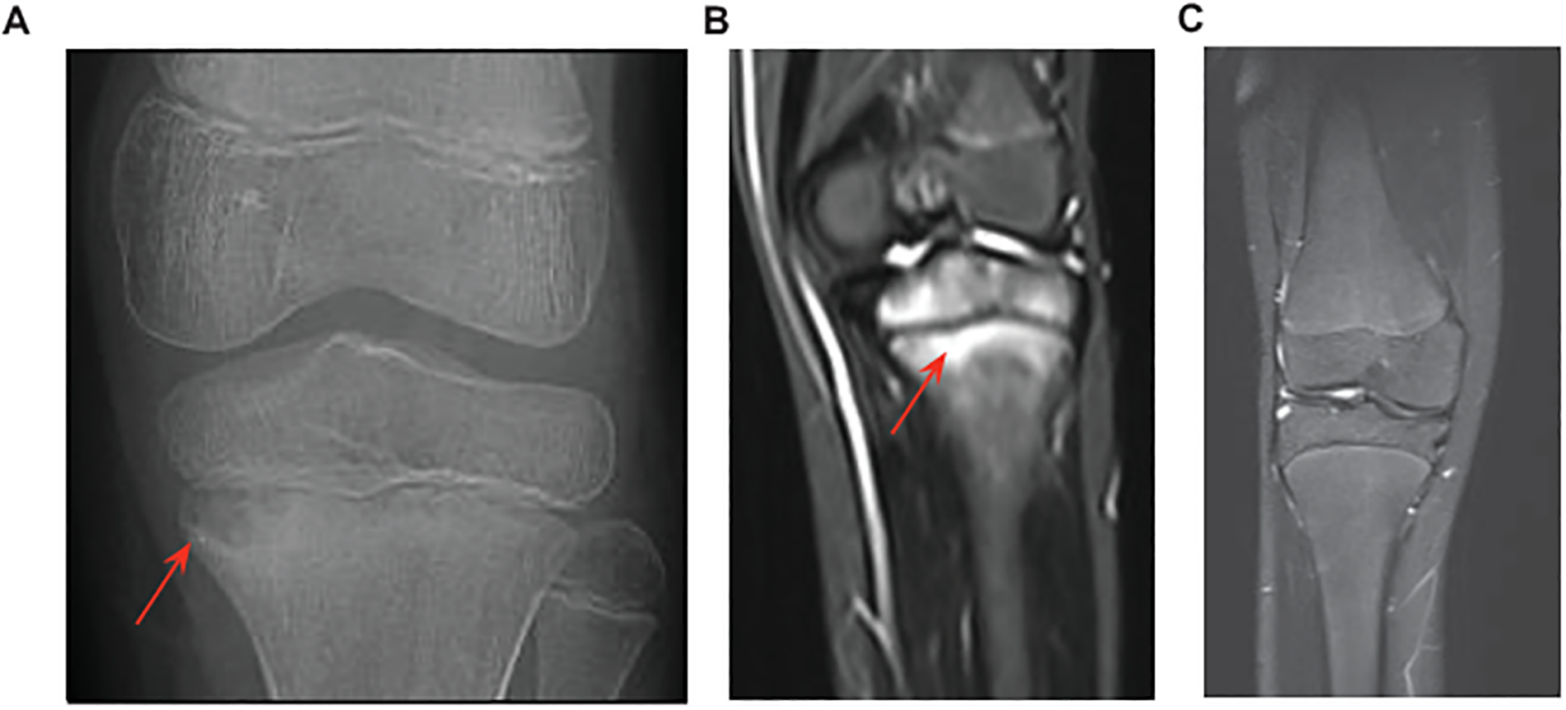
X-ray and MRI knee images from the affected proband. (A) X-ray showing osteomyelitis and a lesion characteristic of CRMO (red arrow) in the left proximal tibia. (B). MRI of the same knee showing inflammation and bone destruction (red arrow). Similar lesions were found in the clavicle, hip, femur, tibia, foot and toes (not shown). (C) MRI of a healthy knee for comparison.
